# Research trends and hotspots in osteoporosis associated with gas molecules and related factors: a bibliometric analysis

**DOI:** 10.4103/mgr.MEDGASRES-D-26-00046

**Published:** 2026-04-11

**Authors:** Yubo Zhao, Yuyan Fan, Yang Yang

**Affiliations:** 1 Department of Human Anatomy, School of Basic Medicine, Shenyang Medical College, Shenyang, Liaoning Province, China; 2 Department of Traditional Chinese Medicine, Beijing Tiantan Hospital, Capital Medical University, Beijing, China; 3 Department of Rehabilitation, The First Hospital of China Medical University, Shenyang, Liaoning Province, China

**Keywords:** bibliometrics, gas molecules, hydrogen, hydrogen sulfide, literature visualization, medical gases, nitric oxide, osteoporosis, oxidative stress, reactive oxygen species, related factors

## Abstract

The pathogenesis of osteoporosis is closely related to the imbalance of oxidative stress. Currently, gas molecules and related factors such as hydrogen sulfide, nitric oxide, and hydrogen have shown great potential in the field of bone metabolism due to their unique redox regulatory properties. However, there is a lack of a systematic review regarding the development trajectory, emerging hotspots, and future trends in this area of research. Using bibliometric analysis methods, this study aims to provide a comprehensive view of the evolution of gas molecules and related factors in osteoporosis research from 2001 to 2025. It clarified the shifting patterns of research hotspots in this field and predicted future development directions. We conducted a literature search in the Web of Science Core Collection database to investigate the role of gas molecules and related factors in osteoporosis research. A total of 387 relevant articles published between 2001 and 2025 were included. We utilized bibliometric tools such as Citespace 6.4.R2, VOSviewer 1.6.20, and Bibliometrix (R-4.5.2) to perform a multidimensional visual analysis of the 387 articles, focusing on publication trends, scientific time span, contributions by countries/institutions, journal distribution, keyword co-occurrence, emerging hotspots, and highly cited papers. We found that: (1) The annual publication volume in this field shows a fluctuating upward trend, peaking in 2024, indicating that the field has entered a relatively mature development phase. (2) China and the United States constitute the first tier, with Harvard University leading globally in publication volume. (3) The most frequently used keywords include “osteoporosis” (112 occurrences), “osteoclasts” (48 occurrences), “osteoblasts” (40 occurrences), “reactive oxygen species” (33 occurrences), “oxidative stress” (31 occurrences), and “nitric oxide” (30 occurrences). (4) Keyword analysis indicates that gas molecules, by regulating the core pathological mechanism of “oxidative stress/reactive oxygen species,” have become a key hub connecting various pathological processes in osteoporosis. Nitric oxide, hydrogen sulfide, and hydrogen represent different stages of development. Nitric oxide is an early classic theme, with a median year of 2012; hydrogen sulfide is a current research hotspot, with a median year of 2020, actively transitioning to therapeutic applications; while hydrogen represents an emerging frontier, with a median year of 2023, indicating the latest exploratory direction. Overall, the research focus on gas molecules and related factors shows a relay-style evolution from nitric oxide to hydrogen sulfide to hydrogen, continuously deepening in conjunction with the core mechanism of “reactive oxygen species/oxidative stress.” (5) The results of the literature analysis indicate that current evidence generally identifies oxidative stress, particularly related to reactive oxygen species, as a key pathological mechanism in the development of osteoporosis, with the nuclear factor erythroid 2-related factor 2-related pathway recognized as an important target for intervention. Inducible nitric oxide synthase produces excessive nitric oxide, which is involved in inflammatory bone loss. Studies from 2001 to 2019 establish nitric oxide as a common mediator of various bone protective agents. Emerging research from 2020 to 2025 demonstrates that hydrogen and hydrogen sulfide have confirmed bone-protective effects, primarily through their strong antioxidant and anti-inflammatory actions, which alleviate oxidative stress damage to bone cells. The recent surge in research on nanocarriers and smart materials is becoming a breakthrough for clinical translation in this field. Findings from this article show that: (1) From 2001 to 2025, the role of gas molecules and related factors in osteoporosis research has transitioned from investigating basic mechanisms to focusing on innovative targeted therapies, with basic research now entering a relatively mature development phase. (2) The most prominent gas molecules and related factors in this field are nitric oxide, hydrogen sulfide, and hydrogen, each representing different stages: exploration of mechanisms, optimization of delivery, and translational application. (3) Future research should focus on the delivery of gas molecules and related factors using new materials while also enhancing studies on the synergistic effects of various gas molecules and the precise spatiotemporal regulation of gas release. This approach will accelerate the clinical translation of gas molecules and related factors in the field of osteoporosis research.

## Graphical abstract

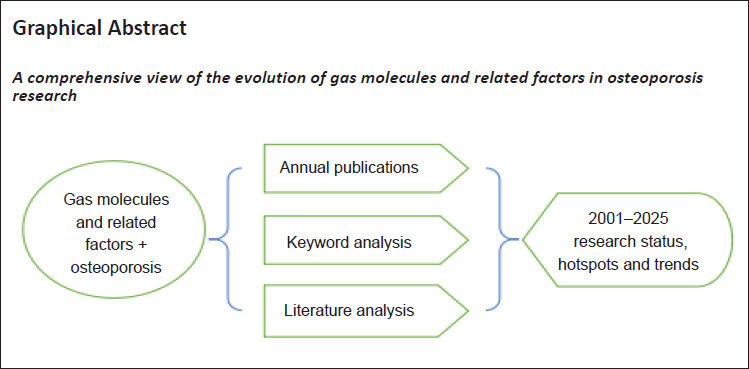

## Introduction

Osteoporosis, characterized by a reduction in bone mass, destruction of bone microstructure, and an increased risk of fractures, has become a major global public health challenge, placing a heavy burden on healthcare systems.^1^,^2^ With the acceleration of population aging, the urgency to develop new prevention and treatment strategies is becoming increasingly evident. Conventional osteoporosis medications, such as bisphosphonates and parathyroid hormone analogs, while effective, have issues related to long-term safety, poor compliance, and variability in individual responses.^1^,^3^,^4^,^5^ In this context, exploring endogenous regulatory mechanisms and developing more targeted novel intervention targets have become forefront directions in this field.

Currently, gas molecules and related factors have shown unique value in the study of bone metabolism regulation. Gas molecules such as nitric oxide (NO), hydrogen sulfide (H_2_S), and hydrogen (H_2_) are involved in the core pathological processes of osteoporosis, such as oxidative stress, inflammatory responses, and cell apoptosis, due to their properties including free diffusion, rapid response, and multi-target regulation.^6^,^7^,^8^,^9^,^10^ Notably, their mechanism of regulating ferroptosis and redox homeostasis via the activation of the nuclear factor erythroid 2-related factor 2 (Nrf2) pathway provides a fresh perspective for understanding the pathogenesis of osteoporosis.^11^ However, current research in this field exhibits significant fragmentation,^6^,^12^,^13^ primarily with the following characteristics: first, studies focus on the isolated effects of single gas molecules, lacking systematic comparisons of the synergistic effects and functional gradients between different gas signaling molecules; second, the technical pathways are dispersed, with a lack of phased organization in translational research from nano-carrier design to smart material applications; third, the evolution of research paradigms is unclear, restricting strategic judgments in transitioning from “phenomenon association” to “targeted intervention.” The gas molecules and related factors discussed in this paper specifically refer to gaseous molecules with signal transduction functions (NO, H_2_S, H_2_, carbon monoxide [CO], oxygen [O_2_]) and their direct derivatives (such as peroxides).

Although numerous experimental studies on gas molecules and related factors and bone metabolism have emerged both domestically and internationally, existing reviews are often limited to conventional qualitative analyses, making it difficult to objectively reveal the dynamic migration trajectory of past research hotspots and future trend predictions.^1^,^14^,^15^,^16^ Bibliometrics, as a quantitative analysis tool based on mathematics and statistics, can comprehensively deconstruct the development context of disciplines using techniques such as knowledge mapping visualization, co-occurrence network analysis, and burst detection.^17^,^18^ Its unique advantage in identifying key nodes in research paradigm transitions and predicting directions for technological breakthroughs aligns perfectly with the current developmental stage of research on gas molecules and related factors as it shifts from basic exploration to clinical translation.^19^,^20^

To offer a comprehensive view of the evolutionary trajectory of gas molecules and related factors in osteoporosis research, the article utilizes bibliometric methods to systematically analyze 387 relevant publications from the Web of Science Core Collection database covering the period from 2001 to 2025. By constructing keyword co-occurrence networks, author collaboration maps, and citation burst analyses, the article elucidates the gradient research patterns associated with NO, H_2_S, and H_2_, alongside their respective developmental stages. It identifies the strategic significance of nanocarriers and smart materials as key technological breakthroughs and highlights the ongoing interest in the Nrf2 pathway as a central intervention target.

The article not only fills the gap of systematic trend analysis in this field but also provides evidence-based decision-making support for researchers to optimize resource allocation and grasp innovation directions, thereby holding significant strategic importance for accelerating the translation and application of gas molecules and related factors from the laboratory to clinical practice.

## Methods

### Literature search

**Figure 1:**
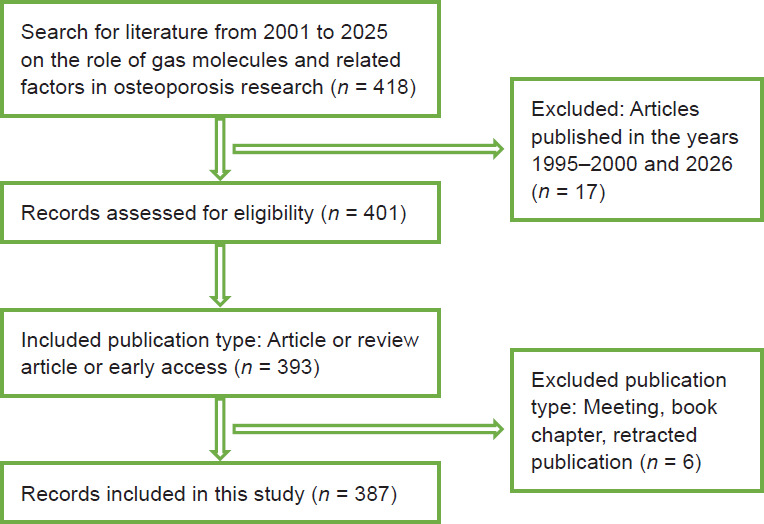
Literature screening flowchart for the Web of Science Core Collection database.

The literature search was conducted with the first author as the searcher. The search date was January 17, 2026. The database used for the search was the Web of Science Core Collection. The inclusion criteria for the literature were as follows: date range from January 1, 2001, to December 31, 2025; types of literature included article, review article, and early access. A total of 387 publications were included in the final analysis. The search strategy is detailed in **Table 1**, and the literature screening process is illustrated in **Figure 1**.

**Table 1:** Literature search strategy (Web of Science Core Collection database)

#	Search query	Search results
1	AB=(Osteoporos* OR Osteopenia* OR Osteoporot* OR “Age-Related Bone Loss*” OR “Perimenopausal Bone Loss*”)	78891
2	TI=(gas OR Gaseous OR Oxygen OR Hypoxia OR “Nitric Oxide” OR “Carbon Dioxide” OR “Hyperbaric Oxygen” OR Ozone OR “Hydrogen peroxide” OR “Carbon Disulfide” OR “Nitrous Oxide” OR “Hydrogen sulfide” OR “Carbon Monoxide” OR “Nitrogen Monoxide” OR “Mononitrogen Monoxide” OR “Sulfur Dioxide” OR “Nitrogen Dioxide” OR Hyperoxia OR Argon OR Helium OR “Compressed Air” OR “Pressurized Air” OR Hydrogen OR Oxide OR Hydride OR Nitrogen OR Methane OR Ethylene OR Ammonia OR Dioxygen OR “Hyperbaric Oxygenation”)	1728053
3	#2 AND #1	418
4	#2 AND #1 AND 1995 OR 1996 OR 1997 OR 1998 OR 1999 OR 2000 OR 2026 (Exclusion - Final Publication Year)	401
5	#2 AND #1 AND 1995 OR 1996 OR 1997 OR 1998 OR 1999 OR 2000 OR 2026 (Exclusion - Final Publication Year) and Article or Review Article or Early Access (Publication Type)	393
6	#2 AND #1 AND 1995 OR 1996 OR 1997 OR 1998 OR 1999 OR 2000 OR 2026 (Exclusion - Final Publication Year) and Article or Review article or Early Access (Publication Type) and Retracted publication or Book chapters or Meeting (Exclusion–Publication Type)	387

### Inclusion and exclusion criteria

Inclusion criteria: (1) Articles focusing on the role of gas molecules and related factors in osteoporosis research; (2) Publication type: Article, review article, and early access; (3) Literature initially published online from 2001 to 2025 and formally published in 2026; (4) Given the central role of oxidative stress, this article will include reactive oxygen species (ROS) as an associated mechanism in the analysis. However, ROS-related literature will not be classified as gas signaling molecules.

Exclusion criteria: Literature classified as meeting, retracted publication, or book chapter.

### Data analysis

#### Data collection and processing

Literature records retrieved from the Web of Science Core Collection database were exported as plain text (“download_XXX.txt”), with each record containing author names, affiliations, publication date, title, abstract, keywords, and other information. Before data analysis, Excel 2019 was used for deduplication, field cleaning, and synonym merging, resulting in a unified analysis dataset of 387 articles. After the screening and processing of these 387 articles, no duplicate literature was found.

#### Data analysis

The article utilizes various mainstream bibliometric and visualization software tools, including Citespace 6.4.R2 Advanced Edition, VOSviewer_1.6.20, the Bibliometrix package (RStudio, R-4.5.2-win), and the bibliometric online tool (https://bibliometric.com/) for multidimensional analysis. The download_XXX.txt file was imported into these software tools for bibliometric analysis.

Citespace 6.4.R2 advanced edition: This software was used to create dual-map overlay visualizations to analyze the flow of knowledge bases and frontiers, revealing the primary disciplines from which knowledge in this field originates (such as molecular biology and immunology) and the main disciplines to which it progresses. Additionally, it extracts data from the Web of Science Core Collection database and identifies highly cited literature.^21^,^22^

VOSviewer 1.6.20: This tool was used to construct keyword co-occurrence networks and density visualizations, allowing for the selection of high-frequency keywords and providing a visual representation of the structure of research themes and hotspot distribution.^23^,^24^

Bibliometrix (R-4.5.2): This R-based package plays a central role in the analysis for this study.^25^ It includes: (1) Descriptive statistics: Calculation of basic bibliometric characteristics, such as annual growth rate, average citations per paper, author collaboration rate. (2) Trend and time span analysis: Fitting annual publication volume curves and cumulative growth curves to predict the development stages of the field. (3) Country and institution analysis: Generating country collaboration network diagrams and bar charts for publication/citation rankings. (4) Journal analysis: Statistics on publication trends of source journals. (5) Keyword analysis: Creating trend graphs for hotspot keywords.

Additionally, the bibliometric online platform (https://bibliometric.com/) was used to support the country analysis.

### Main outcome measures

Using the aforementioned tools, the analysis primarily focused on the following aspects: revealing the overall growth trend and time span stage of research output in the field through annual publication volume and cumulative publication volume curves. The productivity (publication volume) and impact (citation counts) of countries and institutions were analyzed, and their collaboration networks were visualized to identify core research forces. Core research themes were identified through keyword co-occurrence networks. The evolution of research hotspots was tracked using burst detection and thematic trend graphs (such as the shift in research focus from NO to H_2_S and H_2_). By examining classic literature, the article outlines the research focal points and key findings related to various gas molecules and associated factors (such as NO, H_2_S/H_2_, and ROS) across different time periods, thereby validating the trends identified in the keyword analysis.

## Results

### Publication volume and basic information

**Figure 2:**
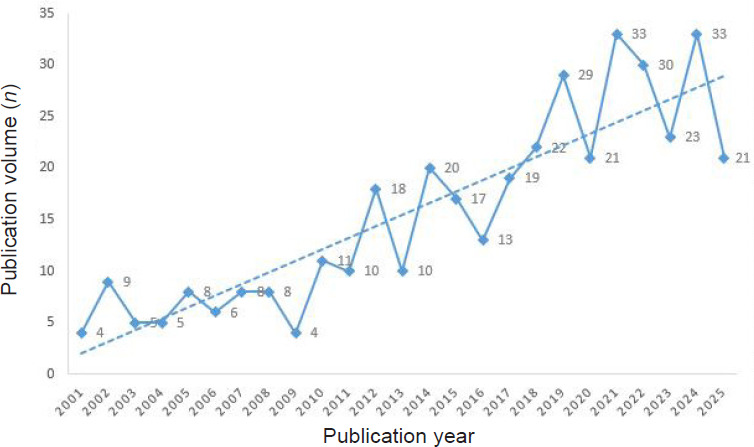
Analysis of volume of included publications.

The publication volume in this field has shown an overall upward trend with fluctuations over the past 25 years. The number of publications increased from 4 in 2001 to 21 in 2025, resulting in a total increase of 17 publications, with an average annual growth of approximately 1.12 publications. There was a noticeable rise in research output after 2012, peaking in 2024, followed by a slight decline in 2025 with 21 publications; however, the overall trend in annual publications remained upward (**Figure 2**).

The 387 publications included in the analysis come from 244 distinct journals. The literature is relatively recent, with an average publication year of around 9 years and an average of 38 citations per paper. Research themes are diverse, featuring over 2000 keywords. A total of 2261 authors contributed to these publications, reflecting a high level of collaboration, with an average of 6.9 authors per publication, of which 20.16% involve international cooperation. The publication types are primarily article (*n* = 350) and review article (*n* = 34), with article being the majority (**Table 2**).

**Table 2:** Key information of included publications

Description	Results
Main information about data	
Time span	2001–2025
Sources (journals)	244
Documents	387
Annual growth rate (%)	7.56
Document average age (year)	9.22
Average citations per document	38
References	16158
Document content	
Keywords plus (ID)	1297
Author keywords (DE)	1020
Authors	
Author counts	2261
Authors of single-authored document	8
Author collaboration	
Single-authored document	9
Co-authors per document	6.9
International co-authorships (%)	20.16
Document type	
Article	350
Article; early access	3
Review	34

### Analysis of the research time span of included publications

**Figure 3:**
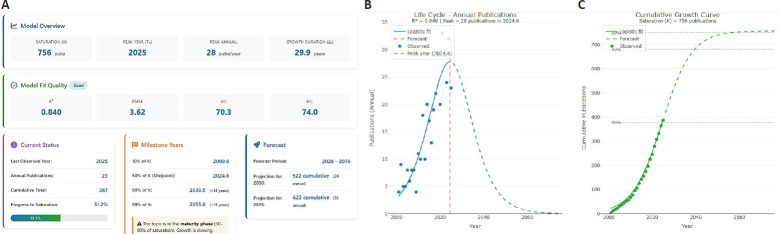
Predictive analysis of the research output–time span model for included publications. (A) Summary of the results from the research topic evolution modeling analysis. The time span analysis is based on a logarithmic growth function, which illustrates the evolution of cumulative publication numbers over time. The topic is currently in a relatively mature development phase, having reached 50–90% saturation, and the growth rate is beginning to decline. The current saturation percentage (K) is categorized as follows: 0–30% indicates the emergence phase or early growth stage; 31–70% corresponds to the rapid growth phase (during which the topic is considered a “hotspot”); 71–90% signifies the later growth phase, approaching a relatively mature development period; and > 90% denotes a relatively mature development phase, nearing saturation. (B) Annual publication time span. The blue solid line represents the logarithmic fit curve for the observed data; the blue dashed line indicates the predicted annual publication count; the blue dots reflect the actual annual publications; and the red dashed vertical line marks the peak year (Tm). (C) Projected future cumulative growth curve over time. The green solid line shows the logistic regression fit for the observed cumulative data; the green dashed line represents the predicted cumulative publication volume; the green dots indicate the observed cumulative publication volume; and the horizontal dashed lines denote the saturation thresholds (50%, 90%).

The total number of publications in this research field has reached 387, accounting for 51.2% of the theoretical saturation point (*n* = 756, **Figure 3A**). The annual publication count displays a bell-shaped curve, with a peak in 2024, and the model’s goodness of fit (*R*²) is 0.840 (**Figure 3B**). The cumulative growth curve reveals an “S” shaped growth pattern, characterized by a slow initial increase, a period of accelerated growth in the mid-phase, and a plateau in the later stages (**Figure 3C**). In summary, this research field has moved beyond the rapid growth phase and is currently approaching its peak. It is expected that the growth rate will gradually decline in the future, ultimately nearing saturation.

### Analysis of publications by country

**Figure 4:**
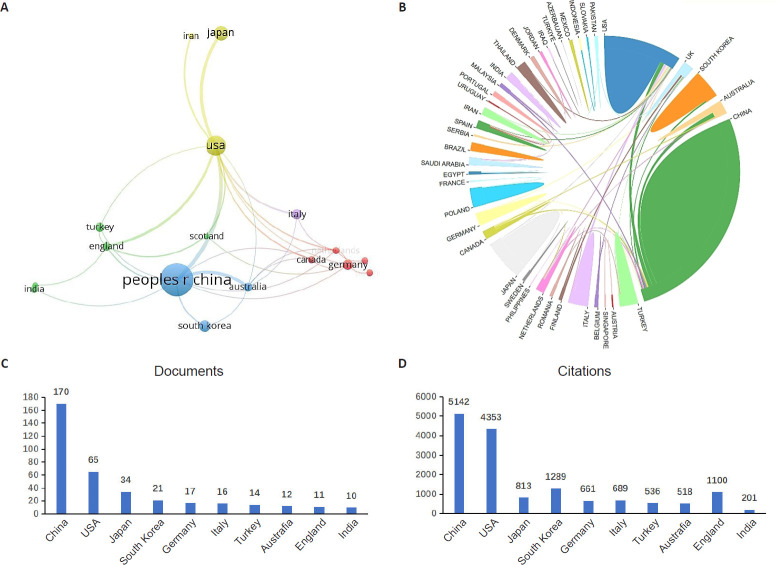
Analysis of publications by country. (A) Co-occurrence network diagram illustrating the collaborative relationships among countries. Different colors represent distinct country clusters, while thicker lines indicate stronger collaborative ties. (B) Sankey diagram depicting national collaboration relationships, with different colors denoting various lines and thicker lines signifying closer collaborations. (C, D) Bar charts showing the top 10 countries based on publication volume and citation counts.

**Figure 4** shows that China and the United States are in the top tier regarding publication volume and total citation count. China leads with 170 publications and 5142 citations, demonstrating a significant advantage in quantity. In contrast, the United States has a much lower publication volume of 65 papers but boasts a total citation count of 4353, indicating a high level of research impact. Together, these two countries account for approximately 45% of the total publication volume and nearly 60% of the total citations, positioning them as key players in this field. However, research from the United States, England, and Scotland appears to be more effective in fostering subsequent academic discussions, suggesting that their findings may be groundbreaking or focused on emerging hotspots in this field.

### Analysis of publications by institute

**Figure 5:**
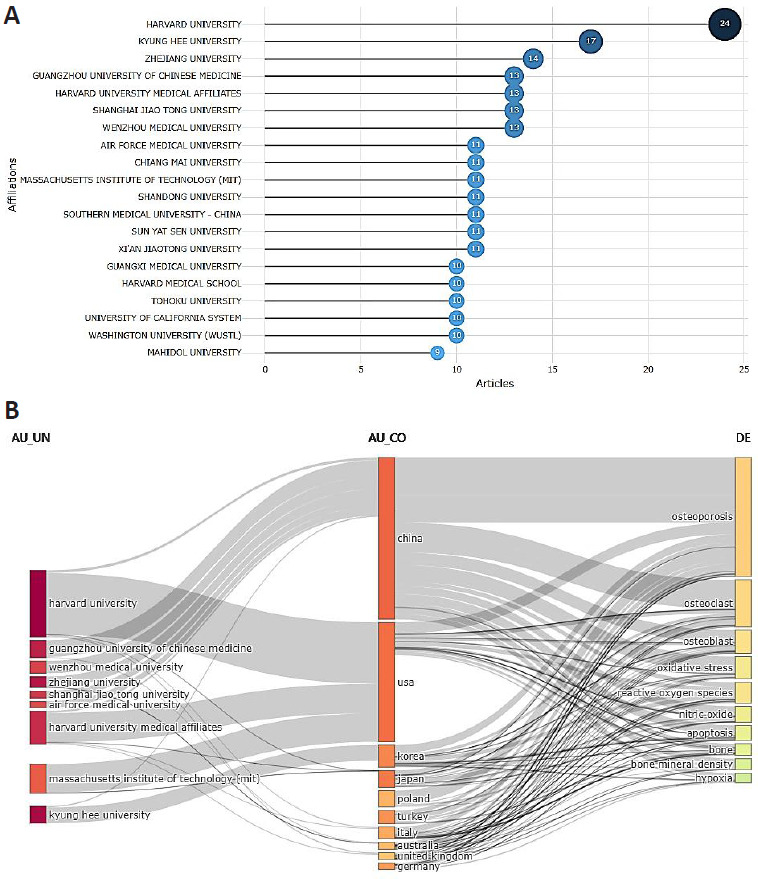
Analysis of publications by institute. (A) Ranking chart of institutions by publication volume. In this chart, larger circles and longer horizontal lines indicate a higher number of publications for each institution. (B) Trend chart illustrating the evolution of institutions (AU_UN), countries (AU_CO), and author keywords (DE). This chart utilizes an interactive Sankey diagram to display the relationships among these three independent dimensions of the literature. The width of each flow (colored band) corresponds to the frequency of co-occurrence between elements; thicker flows signify stronger associations, while thinner flows indicate weaker connections. A denser flow between two elements reflects a higher co-occurrence frequency, suggesting a stronger relationship. The chart demonstrates that institutions from China and the United States lead in research output in this field.

Harvard University leads with 24 publications, and when including its affiliated medical institutions (which account for 13 publications), the total reaches 37 publications, indicating its dominant position and significant research output in this field (**Figure 5A**). Kyung Hee University from South Korea ranks second with 17 publications, followed by the Massachusetts Institute of Technology (MIT) with 11 publications. This indicates strong participation from leading American science and engineering institutions, likely linked to medicine or interdisciplinary studies. Chinese institutions also demonstrate high publication numbers, including Zhejiang University (14 publications), Guangzhou University of Chinese Medicine (13 publications), Shanghai Jiao Tong University (13 publications), Wenzhou Medical University (13 publications), and the Air Force Medical University (11 publications). The primary focus areas for these high-output institutions encompass osteoporosis, osteoblasts, osteoclasts, ROS, NO, and apoptosis (**Figure 5B**).

### Analysis of publications by journal

**Figure 6:**
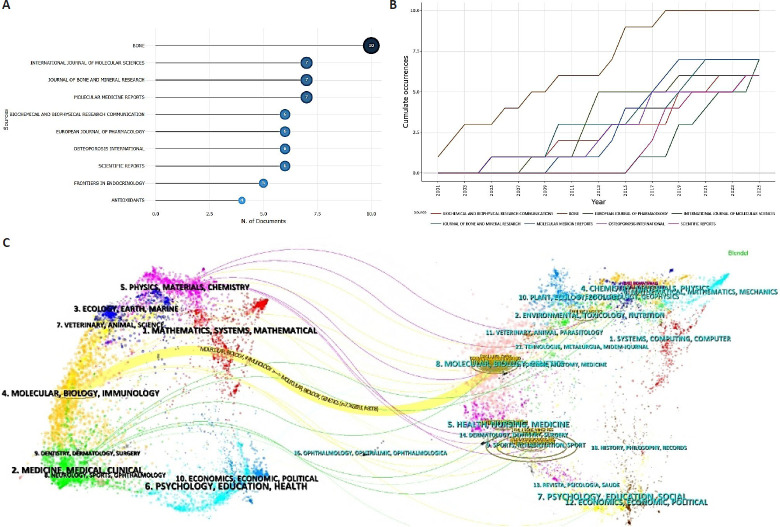
Analysis of publications by journal. (A, B) Total publication volume of the included journals (A) and annual publication trends (B). (C) A dual overlay map of journals created using Citespace 6.4.R2 (advanced version). The left graph illustrates the citing journals (representing knowledge frontiers) while the right graph shows the cited journals (representing the foundational knowledge of the literature). The positioning of the journals is based on their respective disciplinary fields, according to the Journal Citation Reports (JCR) classification from 2011, thereby creating a disciplinary space. The colorful curves in the center illustrate the citation relationships. A curve connecting the cited journal cluster to the citing journal cluster indicates that the latter frequently draws on the knowledge of the former. Journals in the field primarily pertain to molecular biology and immunology, with citations predominantly coming from journals in molecular biology and genetics. This suggests that research publications in the field are largely grounded in molecular biology, evolving from immunology to genetics, with a significant focus on basic research.

The journal *Bone* leads in publication volume, with a total of 10 articles (**Table 3**). *The International Journal of Molecular Sciences, Journal of Bone and Mineral Research*, and *Molecular Medicine Reports* follow closely in second place, each with 7 publications (**Figure 6A**). Between 2001 and 2025, the cumulative publication counts for the journals depicted in **Figure 6B** have increased over time. The overlapping findings among these journals suggest that the research in this field predominantly focuses on molecular biology, immunology, and genetics (**Figure 6C**).

**Table 3:** Key metrics for the top 10 journals by publication volume

Source	Impact factor	h_index	g_index	TC	NP	PY_start
*Bone*	3.6	10	10	582	10	2001
*Journal of Bone and Mineral Research*	5.9	7	7	323	7	2005
*Biochemical and Biophysical Research Communications*	2.2	6	6	191	6	2005
*European Journal of Pharmacology*	4.7	6	6	338	6	2008
*Molecular Medicine Reports*	3.5	6	7	122	7	2010
*International Journal of Molecular Sciences*	4.9	5	7	499	7	2016
*Osteoporosis International*	5.4	5	6	202	6	2005
*Scientific Reports*	3.9	5	6	203	6	2016
*FASEB Journal*	4.2	4	4	103	4	2017
*Frontiers in Endocrinology*	4.6	4	5	33	5	2022

The h-index serves as a metric to evaluate both the volume and impact of academic publications, while the g-index is designed to be more responsive to articles with a high number of citations. TC indicates the total citation count, NP denotes the number of published articles, and PY_start refers to the year when publication commenced.

### Keyword analysis

#### Analysis of keyword co-occurrence centered on core hubs

**Figure 7:**
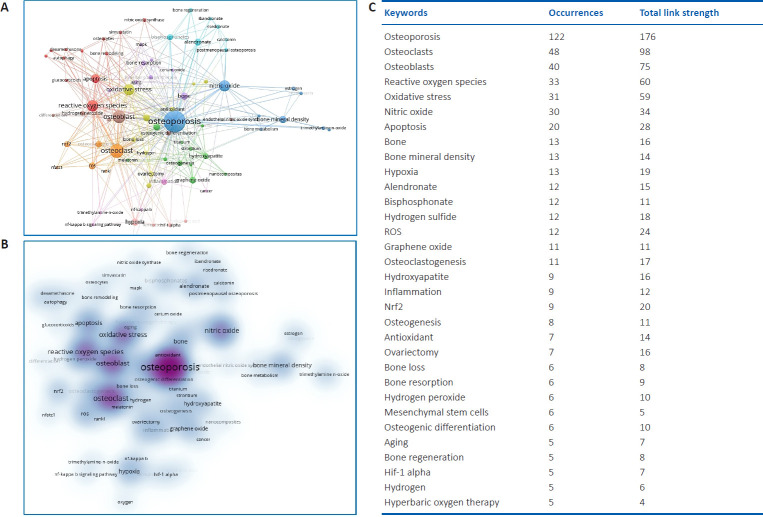
Keyword co-occurrence network of analyzed publications. (A) Keyword visualization network. Nodes of varying colors denote distinct clustering categories; larger nodes signify a higher volume of publications, while thicker lines indicate stronger associations between keywords. The network represents the co-occurrence of keywords that appear at least three times. (B) Keyword density visualization. Darker node colors and larger diameters indicate a greater publication volume associated with each keyword. (C) Statistical chart of keyword frequency and overall connection strength. “Occurrences” refers to how often a keyword appears, whereas “Total Link Strength” denotes the cumulative number of co-occurrences of that keyword with others (including repeated co-occurrences).

**Figure 7** shows that the six most frequently occurring keywords in this field are osteoporosis (112 occurrences), osteoclasts (48 occurrences), osteoblasts (40 occurrences), ROS (33 occurrences), oxidative stress (31 occurrences), and NO (30 occurrences). Among all the keywords, oxidative stress (31 occurrences, with an association strength of 59) and ROS (33 occurrences, with an association strength of 60) are the terms most closely related to the mechanism of “osteoporosis.” This suggests that oxidative damage is a well-recognized fundamental pathological mechanism underlying the disruption of bone homeostasis, specifically the imbalance between osteogenesis and osteoclastogenesis. imbalance of bone homeostasis (the imbalance between osteogenesis and osteoclastogenesis).

The findings presented in **Figure 7** indicate that gas molecules play a crucial role in regulating oxidative stress. This is notably evident in the high frequency and association strength of NO (30 occurrences, with an association strength of 34) and H_2_S (12 occurrences, with an association strength of 18), which are recognized as classic gas molecules and related factors. These gas molecules show a close relationship with antioxidants (7 occurrences, with an association strength of 14) and Nrf2 (9 occurrences, with an association strength of 20), a key transcription factor involved in antioxidant defense. This suggests that research is increasingly focused on how gas molecules influence osteoporosis by modulating redox balance, such as through the activation of the Nrf2 pathway and direct scavenging of ROS. Furthermore, H_2_ (5 occurrences) has emerged as a new medical gas in the field, typically used in the form of H_2_ gas or hydrogen-rich water, and it exerts its effects through selective antioxidant properties, particularly against strong oxidants such as ·OH. Its emergence signifies a broadening of gas treatment methodologies. Thus, gas molecules and related factors, especially NO and H_2_S, have transitioned from emerging roles to becoming central hubs linking oxidative stress, cellular metabolism, inflammation, and apoptosis-related pathological mechanisms in osteoporosis research.

As shown in **Figure 7**, the core conceptual group in the field includes “osteoporosis,” “osteoclasts,” “osteoblasts,” “reactive oxygen species,” and “oxidative stress.” Among these, “oxidative stress/reactive oxygen species” emerges as the mechanism-related keyword most closely linked to “osteoporosis,” highlighting oxidative damage as a fundamental pathological mechanism in the condition. Gas molecules play a crucial role in regulating oxidative stress, as demonstrated by the strong co-occurrence relationships identified between “nitric oxide,” “hydrogen sulfide,” “antioxidants,” and “Nrf2.” The introduction of “hydrogen” signifies a broadening of treatment options. Consequently, gas molecules and related factors have become central hubs that interconnect various pathological mechanisms underlying osteoporosis.

#### Analysis of keyword themes reveals emerging hotspot trends

**Figure 8:**
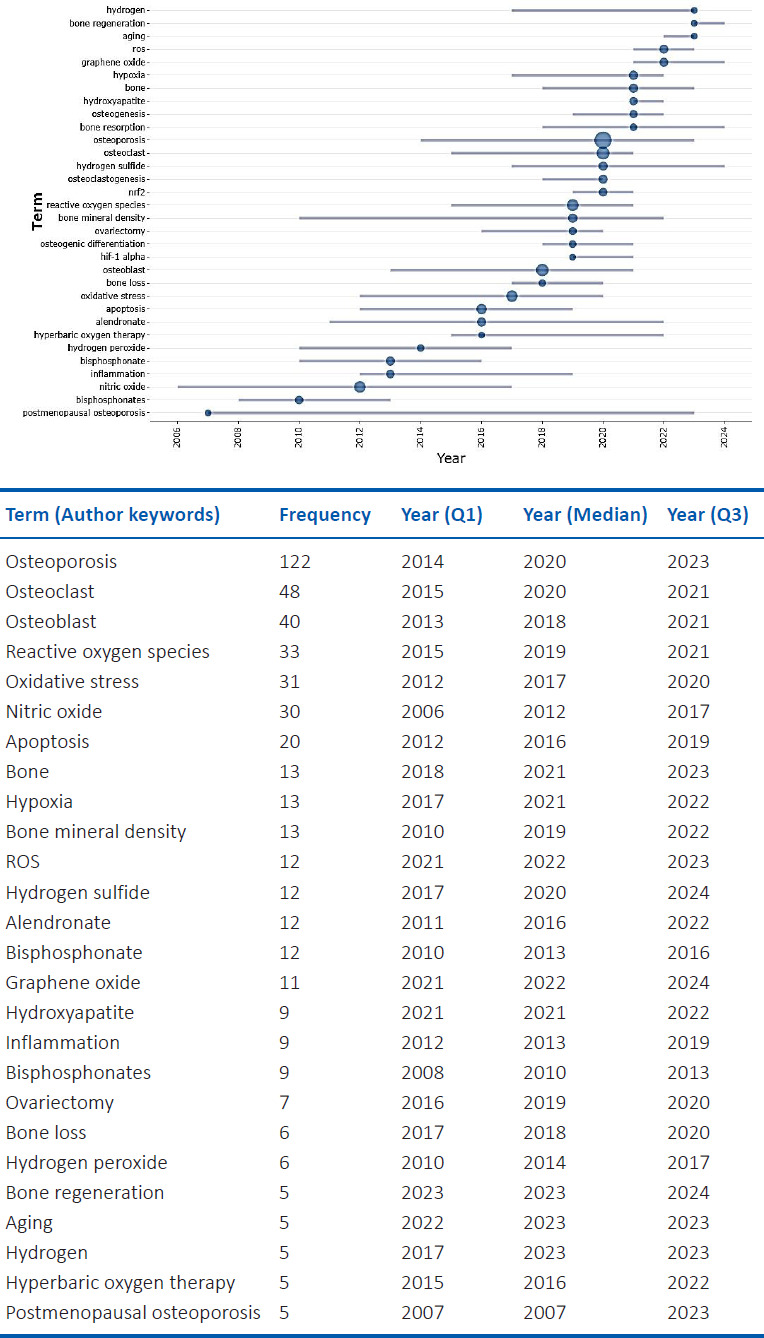
Keyword hotspot trend visualization. The figure illustrates the publication frequency trends for keywords with occurrences of ≥ 5 over the years. In the upper chart, the horizontal line marks the median year (Q1–Q3), while larger circles denote higher frequencies. The lower chart provides statistical data on keyword frequencies and their corresponding years, organized by frequency.

From **Figure 8**, it can be seen that keywords related to gases, gas molecules and related factors, and their associated redox reactions exhibit the following thematic trends:

(1) Characteristics of the NO trend: NO appears prominently, with 30 occurrences; however, its distribution is earlier in timeline, beginning in 2006, with a median publication year of 2012 and Q3 in 2017. This suggests that NO, recognized as one of the earliest gas molecules and related factors, garnered significant attention in osteoporosis research approximately a decade ago, leading to extensive exploration of its regulatory mechanisms in bone resorption and formation. 26

(2) Trend characteristics of H_2_S topics: H_2_S has been recorded 12 times, with its emergence beginning in 2017, a median publication year of 2020, and a projected peak in 2024. This indicates that H_2_S is a recently active and rapidly evolving area of interest. Research has evolved from investigating its mechanisms to focusing on therapeutic applications, such as developing H_2_S delivery systems aimed at bone tissue to enhance its protective effects.^27^,^28^,^29^

(3) Trend characteristics of H_2_ topics: H_2_ has been mentioned 5 times, with its first occurrence in 2017. The median year and Q3 both fall in 2023, suggesting that research on the role of H_2_ in osteoporosis is a rapidly developing area. Initial studies indicate that H_2_ may play a significant role in inhibiting osteoclastogenesis and preserving bone mass, and researchers are starting to investigate combination strategies with other therapies, such as the inhibition of pyroptosis.^30^,^31^

(4) Trend characteristics of ROS/oxidative stress: “Reactive oxygen species” (33 times) and “oxidative stress” (31 times) are both high-frequency keywords. “Reactive oxygen species” (31 times) started in 2015, with a median year of 2019 and Q3 in 2021. Its abbreviation “ROS” (12 times) started in 2021, with a median year of 2022 and Q3 in 2023, indicating a significant recent increase in attention as a specific pathogenic molecule. Related research has clearly revealed that ROS (particularly mitochondrial ROS) are key mechanisms driving osteocyte dysfunction, leading to reduced bone formation and aging-related osteoporosis,^32^ and they have become important therapeutic targets.

(5) Trend characteristics of related microenvironment concepts: The frequency of “hypoxia” (13 occurrences, median year 2021) and “hyperbaric oxygen therapy” (5 occurrences, median year 2016) indicates a notable interest in the regulation of the oxygen microenvironment within bone tissue. This focus is closely tied to the roles of gas molecules and related factors in bone health and pathology.

The figure illustrates the publication frequency trends for keywords with occurrences of ≥ 5 over the years. In the upper chart, the horizontal line marks the median year (Q1–Q3), while larger circles denote higher frequencies. The lower chart provides statistical data on keyword frequencies and their corresponding years, organized by frequency.

In summary, NO is identified as an “early classic” theme, exhibiting a high frequency of occurrence with a median year of 2012, indicating that its primary academic contributions were made approximately a decade ago. H_2_S is classified as a “current hotspot,” indicating a moderate frequency and a more recent median year of 2020, with a Q3 approaching the present (2024), suggesting active research that is shifting toward therapeutic applications. H_2_ is characterized as an “emerging frontier,” with a lower frequency but the most recent median year of 2023, marking it as a cutting-edge area of exploration. ROS and oxidative stress remain central mechanisms driving the evolution of research focus. Overall, the study of gas molecules and related factors reflects a progressive development trajectory from NO (classic) to H_2_S (hotspot), and subsequently to H_2_ (frontier), aligning with the deeper investigation of the core mechanisms associated with reactive oxygen.

### Analysis of literature hotspots

Based on the aforementioned keywords, this article presents the primary functions of gas molecules and related factors in osteoporosis research, including ROS, NO, H_2_, and H_2_S. The frequency of their citations serves as an indicator of the current academic interest in these topics.

#### Analysis of highly cited literature on nitric oxide and osteoporosis

The research directions of highly cited literature on NO and osteoporosis can be divided into two main stages, as shown in **Table 4**.

**Table 4:** Highly cited literature on nitric oxide and osteoporosis (sorted by citation frequency)

Authors	Hot issues	Article title	Source journal	Document type	Times cited in Web of Science core collection	Publication year
Ozgocmen et al.^33^	Oxidative stress markers as therapeutic targets for postmenopausal osteoporosis.	Role of antioxidant systems, lipid peroxidation, and NO in postmenopausal osteoporosis	*Molecular and Cellular Biochemistry*	Article	167	2007
Armour et al.^34^	Targeting the iNOS pathway to reduce inflammatory bone loss	Activation of the inducible NO synthase pathway contributes to inflammation-induced osteoporosis by suppressing bone formation and causing osteoblast apoptosis	*Arthritis and Rheumatism*	Article	86	2001
Ye et al.^10^	Near-infrared light-triggered upconversion nanoplatform for precise NO delivery	Near-infrared light and upconversion nanoparticle defined NO-based osteoporosis targeting therapy	*ACS Nano*	Article	80	2021
Hukkanen et al.^35^	Nitroglycerin as a NO donor in the treatment of estrogen deficiency-induced osteoporosis	Effect of NO donor nitroglycerin on bone mineral density in a rat model of estrogen deficiency-induced osteopenia	*Bone*	Article	56	2003
Bakker et al.^36^	Collaborative regulation of NO production in bone cells by estrogen and mechanical loading	Additive effects of estrogen and mechanical stress on NO and prostaglandin E2 production by bone cells from osteoporotic donors	*Osteoporosis International*	Article	55	2005
Ozgocmen et al.^37^	Effects of anti-osteoporosis drugs on redox status	Effects of calcitonin, risedronate, and raloxifene on erythrocyte antioxidant enzyme activity, lipid peroxidation, and NO in postmenopausal osteoporosis	*Archives of Medical Research*	Article	53	2007
Das^38^	NO as a common signaling pathway for various bone protectors	NO as the mediator of the antiosteoporotic actions of estrogen, statins, and essential fatty acids	*Experimental Biology and Medicine*	Review	51	2002
Yin et al.^39^	Statins protect bone by antioxidant action and restoring NO pathways	Protection against osteoporosis by statins is linked to a reduction of oxidative stress and restoration of NO formation in aged and ovariectomized rats	*European Journal of Pharmacology*	Article	50	2012
Wimalawansa^40^	NO replacement therapy as an alternative to estrogen therapy	NO: novel therapy for osteoporosis	*Expert Opinion on Pharmacotherapy*	Review	43	2008
Carradori et al.^41^	Dual target therapeutic strategy of NO donors and carbonic anhydrase inhibitors	NO donors and selective carbonic anhydrase inhibitors: A dual pharmacological approach for the treatment of glaucoma, cancer and osteoporosis	*Molecules*	Review	41	2015
Oktem et al.^42^	Melatonin protects bone by regulating iNOS expression and cell apoptosis	Evaluation of the relationship between inducible nitric oxide synthase (iNOS) activity and effects of melatonin in experimental osteoporosis in the rat	*Surgical and Radiologic Anatomy*	Article	35	2006
Lee et al.^43^	Bionic NO-releasing scaffold regulates bone homeostasis and promotes regeneration	NO-releasing bioinspired scaffold for exquisite regeneration of osteoporotic bone via regulation of homeostasis	*Advanced Science*	Article	34	2023
Jeddi et al.^44^	Role of reduced bioavailability of NO in diabetic osteoporosis	Role of NO in type 1 diabetes-induced osteoporosis	*Biochemical Pharmacology*	Review	19	2022
Jamal and Hamilton^45^	Clinical potential of nitrate-based NO donors in reducing fracture risk	NO donors for the treatment of osteoporosis	*Current Osteoporosis Reports*	Article	18	2012

NO: Nitric oxide.

(1) Previous classic research hotspots (2001–2019)

The highly cited publications during this period established the theoretical foundation for the role of NO in bone biology, primarily focusing on several core pathways.

Role of NO and oxidative stress in postmenopausal osteoporosis: Early research has established a link between oxidative stress and osteoporosis, highlighting changes in NO levels as significant indicators. For instance, a study by Ozgocmen et al.^33^ (2007), which has been cited 167 times, found that postmenopausal women with osteoporosis showed decreased activity of erythrocyte antioxidant enzymes, elevated levels of lipid peroxidation products, and increased NO levels, all of which correlated with bone density. Furthermore, Ozgocmen et al.^37^ (2007), cited 53 times, proposed that NO acts as a common signaling pathway for various bone-protective agents, suggesting that markers of oxidative stress could serve as crucial indicators of bone loss in postmenopausal women.

Key role of inducible NO synthase in inflammatory bone loss: Armour et al.^34^ (2001), cited 86 times, used a knockout mouse model lacking the inducible nitric oxide synthase gene to reveal, for the first time, that the activation of the inducible nitric oxide synthase pathway contributes to inflammation-induced osteoporosis. Their findings indicate that this pathway inhibits bone formation and triggers apoptosis in osteoblasts. This study offers valuable insights into the mechanisms underlying bone loss in inflammatory conditions, such as rheumatoid arthritis.

NO as a common mediator of the anti-osteoporotic effects of estrogen and statins: Das^38^ (2002, cited 51 times) presented a comprehensive viewpoint in his review, proposing that a variety of structurally distinct drugs, including estrogen, statins, and essential fatty acids, may promote their anti-osteoporotic effects through the enhancement of endogenous NO production. This hypothesis connects several therapeutic pathways to NO signaling, carrying significant implications for treatment strategies.

Bone protective effects of NO donors (e.g., nitroglycerin): Based on the theory of NO-mediated bone protection, researchers started investigating the therapeutic potential of nitric oxide donors. Hukkanen et al.^35^ (2003, cited 56 times) demonstrated in an ovariectomized rat model that nitroglycerin effectively mitigated the loss of bone density associated with estrogen deficiency, showing effects similar to those of estrogen. This finding provides preclinical support for the application of NO replacement therapy.

(2) Emerging research hotspots in recent years (2020–2025)

In recent years, prominent research frontiers have focused on addressing therapeutic challenges such as the short half-life and restricted diffusion radius of NO. This has led to an emphasis on creating precise and controllable systems for NO delivery.

NO targeted therapy driven by nanotechnology: This represents the most dynamic area of research at present. Investigations are concentrated on developing bone-targeted nanoplatforms designed to facilitate the controlled release of NO in specific local bone regions. For instance, Ye et al.^10^ (2021, cited 80 times) created a system utilizing upconversion nanoparticles that respond to near-infrared light, enabling precise regulation of NO donor release. Their studies demonstrated effective bone targeting and significant anti-osteoporotic effects in both *in vivo* and *in vitro* settings.

Multifunctional biomimetic scaffolds for osteoporotic bone regeneration: Incorporating NO release functionality into bone tissue engineering scaffolds facilitates a synergistic approach to “regulating bone homeostasis.” Lee et al.^43^ (2023, cited 34 times) described a biomimetic scaffold that not only releases NO but also incorporates additional active factors. This innovative design achieved remarkable bone regeneration in a rat model of osteoporosis by effectively modulating the balance between osteoblasts and osteoclasts.

Mechanistic insights into NO-induced osteoporosis in specific diseases (such as diabetes): Recent reviews have explored the role of NO in various pathological conditions in greater depth. For instance, Jeddi et al.^44^ (2022, cited 19 times) provided a systematic analysis of how reduced NO bioavailability contributes to osteoporosis induced by type 1 diabetes. They also discussed the potential of inorganic nitrates and nitrites as innovative NO donor therapies.

Dual or multiple target drug design: The integration of NO donors with other therapeutic mechanisms, such as carbonic anhydrase inhibition, has led to the development of multifunctional molecules. Carradori et al.^41^ (2015, cited 41 times) reviewed the dual pharmacological approach of pairing NO donors with carbonic anhydrase inhibitors, emphasizing the active research into combination therapy strategies for the treatment of osteoporosis. Consequently, traditional research hotspots aim to uncover the fundamental mechanisms of NO in the pathophysiology of osteoporosis, especially its interactions with oxidative stress, inflammation, and hormonal factors. In contrast, emerging research hotspots are focused on technological advancements, leveraging nanotechnology, tissue engineering, and smart materials to enable precise and targeted delivery of NO. This shift aims to cultivate the next generation of safer and more effective treatment strategies for osteoporosis

#### Analysis of highly cited literature on H_2_, hydrides, and osteoporosis

Specific highly cited literature related to H_2_, hydrides, and osteoporosis is listed in **Table 5**.

**Table 5:** High-impact literature on hydrogen, hydrides, and osteoporosis (sorted by citation frequency)

Authors	High-impact literature	Article title	Source journal	Times cited in Web of Science Core Collection	Publication year
Baek et al.^46^	Exploration of the population association between oxidative stress and osteoporosis and the mechanisms of osteoclasts	Association of oxidative stress with postmenopausal osteoporosis and the effects of hydrogen peroxide on osteoclast formation in human bone marrow cell cultures	*Calcified Tissue International*	266	2010
Xu et al.^47^	Protective effects and mechanisms of H_2_S on osteoblasts	Hydrogen sulfide protects MC3T3-E1 osteoblastic cells against H_2_O_2_-induced oxidative damage-implications for the treatment of osteoporosis	*Free Radical Biology and Medicine*	159	2011
Zhou et al.^48^	Injection-injectable hydrogen-releasing hydrogel for osteoporotic bone repair	An injectable magnesium-loaded hydrogel releases hydrogen to promote osteoporotic bone repair via ROS scavenging and immunomodulation	*Theranostics*	55	2024
Guo et al.^49^	Verification of the efficacy of hydrogen water in preventing osteoporosis in animal models	Hydrogen water consumption prevents osteopenia in ovariectomized rats	*British Journal of Pharmacology*	34	2013
Kruppke et al.^50^	Study on the osteogenic activity of strontium ion-doped biomaterials	Gelatin-modified calcium/strontium hydrogen phosphates stimulate bone regeneration in osteoblast/osteoclast co-culture and in osteoporotic rat femur defects-*in vitro* to *in vivo* translation	*Molecules*	18	2020
Hao et al.^51^	Clinical association between serum H_2_S levels and bone density in patients with osteoporosis	Association of hydrogen sulfide with femoral bone mineral density in osteoporosis patients: a preliminary study	*Medical Science Monitor*	12	2021
Wu et al.^31^	NF-κB mechanism by which hydrogen inhibits osteoclast differentiation	Hydrogen gas protects against ovariectomy-induced osteoporosis by inhibiting NF-κB activation	*Menopause*	12	2019
Qin et al.^52^	Bone-targeted H_2_S nanodelivery system for the treatment of osteoporotic fractures	A bone-targeting hydrogen sulfide delivery system for treatment of osteoporotic fracture via macrophage reprogramming and osteoblast-osteoclast coupling	*Advanced Functional Materials*	8	2025
Liu et al.^53^	Bone-targeted hydrogen-loaded nanocomposites for remodeling the osteoporotic microenvironment	Palladium-based nanocomposites remodel osteoporotic microenvironment by bone-targeted hydrogen enrichment and zincum repletion	*Research*	7	2024
Jiang et al.^7^	Reactive oxygen species-responsive H_2_S-releasing hydrogel regulates bone immune balance	Harnessing hydrogen sulfide in injectable hydrogels that guide the immune response and osteoclastogenesis balance for osteoporosis treatment	*Materials Today Bio*	6	2024
Carnovali et al.^54^	Specific inhibition of osteoclasts by molecular hydrogen in a zebrafish model	Molecular hydrogen prevents osteoclast activation in a glucocorticoid-induced osteoporosis zebrafish scale model	*Antioxidants*	6	2023

H_2_S: Hydrogen sulfide; NF-κB: nuclear factor kappa-light-chain -enhancer of activated B cells.

(1) Previous classic research hotspots (2001–2019)

Baek et al.^46^ (2010, cited 266 times) were the first to confirm, through a population study, that the serum oxidative stress marker 8-OH-dG is negatively correlated with bone density in postmenopausal women. They also discovered that H_2_O_2_ can promote the formation of human bone marrow-derived osteoclasts by upregulating RANKL/M-CSF. This finding directly establishes a causal link between “oxidative stress–increased bone resorption–bone loss,” marking a significant milestone in the field. In a subsequent study, Xu et al.^47^ (2011, cited 159 times) systematically reported for the first time that hydrogen sulfide (H_2_S) can protect osteoblasts (MC3T3-E1) from oxidative damage and apoptosis induced by H_2_O_2_, while also promoting their proliferation and differentiation, with mechanisms involving the p38/ERK MAPK pathway. This opened a new research avenue regarding the protective role of H_2_S in bone metabolism and clarified its cellular protective mechanisms. Additionally, Guo et al.^49^ (2013, cited 34 times) were the first to show in an ovariectomized rat model that long-term consumption of hydrogen-rich water can effectively prevent bone loss, enhance bone microstructure and mechanical properties, with effects linked to reductions in oxidative stress and inflammation. This provides crucial preclinical evidence supporting H_2_ therapy for osteoporosis.

(2) Emerging research hotspots in recent years (2020–2025)

Zhou et al.^48^ (2024), cited 55 times, developed an injectable magnesium-loaded hydrogel (Mg@PEG-PLGA) designed to release H_2_ at bone defect sites. This innovative material functions by scavenging ROS, promoting macrophage polarization towards the M2 phenotype, inhibiting the NF-κB pathway, and enhancing osteogenesis while suppressing osteoclastogenesis. As a result, it aids in the repair of osteoporotic bone defects, representing a cutting-edge approach that merges H_2_-releasing materials with immune microenvironment regulation. In another study, Qin et al.^52^ (2025), cited 8 times, created a bone-targeted zeolitic imidazolate framework (ZIF)-based H_2_S delivery system (ZIF-H_2_S-SDSSD). This system enables precise release of H_2_S and zinc ions (Zn^2+^) within bone tissue, effectively reprogramming macrophage metabolism and regulating the coupling between osteoclasts and osteoblasts. This approach promotes fracture healing and mitigates osteoporosis, highlighting the significant potential of a new generation of bone-targeted nanomedicines for the systemic treatment of osteoporotic fractures. Additionally, Liu et al.^53^ (2024), cited 7 times, developed a bone-targeted palladium-based nanocomposite [A-Z@Pd(H)], which integrates H_2_-loaded nanomaterials for ROS scavenging with a zinc-releasing framework. This composite remodels the osteoporotic microenvironment through immune modulation and enhances osteogenesis. This indicates a comprehensive strategy for the synergistic treatment of both H_2_ therapy and ion therapy.

In summary, research in this field has progressed from the initial validation of the fundamental role of oxidative stress to a detailed investigation of the specific molecular mechanisms associated with H_2_ and H_2_S. This evolution has led to the development of engineered, targeted therapeutic materials. Classic studies have laid the theoretical foundation, while the latest research focuses on addressing the clinical translation challenges of safely, effectively, and precisely delivering these gas molecules to sites of bone lesions.

#### Reactive oxygen species and their signaling molecules in osteoporosis

A thorough examination of highly cited literature (typically those with over 30 citations) related to ROS and their signaling molecules in osteoporosis reveals several common themes. Research consistently demonstrates that the excessive buildup of ROS is a significant pathological factor contributing to osteoporosis. This accumulation can hinder osteoblast differentiation, enhance osteoclastogenesis, and trigger apoptosis in osteocytes. Further studies have clarified the classical signaling pathways through which ROS influence bone metabolism, primarily including: (1) the RANKL/RANK/NF-κB pathway, which is central to the regulation of osteoclast differentiation; (2) MAPK pathways (such as p38, ERK, and JNK), which serve as mediators for cellular stress and differentiation signals; (3) NFATc1 signaling, a crucial transcription factor involved in osteoclastogenesis; and (4) the Nrf2/HO-1 antioxidant pathway, an essential defense mechanism that aids cells in combating oxidative stress.

Specific highly cited literature is listed in **Table 6**.

**Table 6:** Recent advances in reactive oxygen species and signaling molecules in osteoporosis research (sorted by citation frequency)

Authors	Hot issues	Article title	Source journals	Document type	Times cited in Web of Science Core Collection	Publication year
Tan et al.^55^	ROS mediate the decline in osteogenic differentiation capacity of adult rat mesenchymal stem cells under cyclic mechanical stretching, potentially representing a mechanism underlying age-related osteoporosis.	Decreased osteogenesis of adult mesenchymal stem cells by reactive oxygen species under cyclic stretch: a possible mechanism of age-related osteoporosis	*Bone Research*	Article	94	2015
Huang et al.^56^	The Chinese herbal compound gastrodin exerts anti-osteoporotic effects by reducing ROS through its antioxidant activity, thereby promoting osteogenesis while inhibiting adipogenesis and osteoclastogenesis.	Gastrodin: an ancient Chinese herbal medicine as a source for anti-osteoporosis agents via reducing reactive oxygen species	*Bone*	Article	74	2015
Qin et al.^57^	The ethnic medicine Osteoking treats osteoporosis by alleviating oxidative stress and lowering ROS levels.	Anti-osteoporosis effects of osteoking via reducing reactive oxygen species	*Journal of Ethnopharmacology*	Article	67	2019
Hong et al.^58^	The novel flavonoid glycoside Robinin inhibits osteoclast formation by targeting RANKL, suppressing NFATc1 signaling pathways, and reducing ROS production.	A novel RANKL-targeted flavonoid glycoside prevents osteoporosis through inhibiting NFATc1 and reactive oxygen species	*Clinical and Translational Medicine*	Article	61	2021
Yu et al.^59^	A novel polysaccharide-coated iron oxide nanoparticle (Fe_2_O_3_@PSC) was developed to simultaneously supplement iron and scavenge excess ROS, thereby preventing iron-accumulation-related osteoporosis.	Development of a novel polysaccharide-based iron oxide nanoparticle to prevent iron accumulation-related osteoporosis by scavenging reactive oxygen species	*International Journal of Biological Macromolecules*	Article	44	2020
Chen et al.^60^	The traditional Chinese medicine compound Notopterol reduces ROS by inhibiting the RANKL signaling pathway and activating the Nrf2 antioxidant pathway, thereby suppressing osteoclastogenesis.	Notopterol attenuates estrogen deficiency-induced osteoporosis via repressing RANKL signaling and reactive oxygen species	*Frontiers in Pharmacology*	Article	42	2021
Huang et al.^61^	Myricitrin protects osteoblast differentiation under oxidative stress by reducing ROS and bone resorption factor expression.	Protective effects of myricitrin against osteoporosis via reducing reactive oxygen species and bone-resorbing cytokines	*Toxicology and Applied Pharmacology*	Article	37	2014
Mu et al.^62^	Combined total flavonoids from *Cynanchum wilfordii* and calcium carbonate alleviate osteoporosis by decreasing ROS production, enhancing antioxidant capacity, and activating the PI3K/Akt/mTOR pathway.	Total flavonoids of Rhizoma Drynariae combined with calcium attenuate osteoporosis by reducing reactive oxygen species generation	*Experimental and Therapeutic Medicine*	Article	30	2021
Ran et al.^63^	Cadmium exposure induces osteoclast apoptosis by regulating p53 acetylation through the ROS/SIRT1/PGC-1α pathway, leading to osteoporosis.	Reactive oxygen species control osteoblast apoptosis through SIRT1/PGC-1α/p53Lys382 signaling, mediating the onset of Cd-induced osteoporosis	*Journal of Agricultural and Food Chemistry*	Article	29	2023
Ye et al.^64^	An ROS-scavenging hydrogel loaded with MnO_2_ nanoparticles improves the osteoporotic microenvironment, protects transplanted mesenchymal stem cells, and promotes bone healing.	Reactive oxygen species scavenging hydrogel regulates stem cell behavior and promotes bone healing in osteoporosis	*Tissue Engineering and Regenerative Medicine*	Article	20	2023
Wang et al.^65^	Teriparatide suppresses osteocyte apoptosis by activating the AKT pathway to reduce glucocorticoid-induced ROS levels.	Role of teriparatide in glucocorticoid-induced osteoporosis through regulating cellular reactive oxygen species	*Orthopaedic Surgery*	Article	20	2018
Liu et al.^66^	6′-O-Galloylpaeoniflorin mitigates osteoclastogenesis by inhibiting ROS and downstream MAPKs/c-Fos/NFATc1 signaling pathways.	6′-O-Galloylpaeoniflorin attenuates osteoclasto-genesis and relieves ovariectomy-induced osteoporosis by inhibiting reactive oxygen species and MAPKs/c-Fos/NFATc1 signaling pathway	*Frontiers in Pharmacology*	Article	19	2021
Pan et al.^67^	The antihypertensive agent azilsartan inhibits osteoclastogenesis by activating Nrf2 signaling, reducing ROS production, and suppressing MAPK/NF-κB pathways.	Azilsartan suppresses osteoclastogenesis and ameliorates ovariectomy-induced osteoporosis by inhibiting reactive oxygen species production and activating Nrf2 signaling	*Frontiers in Pharmacology*	Article	18	2021
Chen et al.^68^	Develop an ROS-responsive bionic multilayer titanium implant for *in situ* delivery of α-MSH to regulate bone remodeling balance in osteoporotic conditions.	Construction of a reactive oxygen species-responsive biomimetic multilayered titanium implant for *in situ* delivery of α-melanocyte-stimulating hormone to improve bone remolding in osteoporotic rats	*Applied Materials Today*	Article	9	2021
Xu et al.^69^	The ginger compound cedrol inhibits osteoclastogenesis by reducing ROS levels and blocking NFATc1, NF-κB, and MAPK signaling pathways.	Cedrol, a Ginger-derived sesquiterpineol, suppresses estrogen-deficient osteoporosis by intervening NFATc1 and reactive oxygen species	*International Immunopharmacology*	Article	8	2023
Wong et al.^70^	The furanquinoline alkaloid dictamnine targets RANKL to inhibit osteoclastogenesis by suppressing NF-κB signaling and reducing ROS.	A novel rankl-targeted furoquinoline alkaloid ameliorates bone loss in ovariectomized osteoporosis through inhibiting the NF-κB signal pathway and reducing reactive oxygen species	*Oxidative Medicine and Cellular Longevity*	Article	6	2022
Yuan et al.^71^	The fungicide propypiramide disrupts ROS production, leading to notochord malformations, craniofacial abnormalities, and osteoporosis in zebrafish.	Propineb induced notochord deformity, craniofacial malformation, and osteoporosis in zebrafish through dysregulated reactive oxygen species generation	*Aquatic Toxicology*	Article	3	2023
Qiu et al.^72^	Danphenolic acid A suppresses ROS by activating the Nrf2-HO-1 antioxidant pathway, thereby blocking NF-κB/MAPK/NFATc1 signaling and inhibiting osteoclast activity.	Salvianolic acid a mitigates osteoporotic bone loss by repressing reactive oxygen species via the Nrf2-HO-1 pathway	*Phytotherapy Research*	Article	1	2025
Shi et al.^73^	A bone-targeted platinum nanoframework was developed to dual-inhibit osteoclasts by simultaneously blocking the TMEM16A ion channel and scavenging ROS.	Bone-targeted platinum nanoframework prevents postmenopausal osteoporosis via transmembrane protein 16A channel blockade and reactive oxygen species scavenging	*ACS Nano*	Article; Early Access	0	Online pre-publication in 2025, official publication in 2026
Han et al.^74^	In high-glucose environments, ROS cause osteocyte dysfunction by activating mTOR, impairing autophagy, and inducing apoptosis.	Reactive oxygen species (ROS) drive osteocyte dysfunction in diabetic osteoporosis by impairing autophagy and triggering apoptosis	*Antioxidants*	Article	0	2025

α-MSH: Alpha-melanocyte-stimulating hormone; MAPK/NF-κB: mitogen-activated protein kinase/nuclear factor kappa-light-chain-enhancer of activated B cells; MAPKs/c-Fos/NFATc1: mitogen-activated protein kinases/c-Fos/nuclear factor of activated T-cells, cytoplasmic; Nrf2-HO-1: nuclear factor erythroid 2-related factor 2/heme oxygenase 1; PI3K/Akt/mTOR: phosphoinositide 3-kinase/protein kinase B/mammalian target of rapamycin; ROS: reactive oxygen species; ROS/SIRT1/PGC-1α: reactive oxygen species/Sirtuin 1/peroxisome proliferator-activated receptor gamma coactivator 1-alpha.

(1) Classic research hotspots (2019–2021)

Tan et al.^55^ (2015, cited 94 times): This study was the first to uncover the mechanisms underlying age-related osteoporosis in the context of mechanical stimulation. The researchers discovered that mesenchymal stem cells from adult rats produced higher levels of ROS when subjected to periodic stretching compared to their younger counterparts. Additionally, there was a downregulation of superoxide dismutase 1, which resulted in a reduced osteogenic capacity. Notably, the antioxidant N-acetylcysteine was found to reverse these effects. Huang et al.^56^ (2015, cited 74 times): This investigation exemplifies the study of the anti-osteoporotic properties of traditional Chinese medicine monomers. It systematically demonstrated that Gastrodin lowers reactive oxygen levels, enhances osteogenic differentiation, and inhibits osteoclastogenesis, ultimately leading to improved bone microstructure in an ovariectomized mouse model.

(2) Emerging research hotspots (2020–2025)

The research trends in the field from 2020 to 2025 can be summarized as follows: a) In-depth mechanism exploration: Studies are increasingly focusing on detailed molecular mechanisms. For instance, Ran et al.^63^ (2023, cited 29 times) identified that cadmium exposure leads to mitochondrial dysfunction and osteoblast apoptosis through the ROS/SIRT1/PGC-1α/p53 signaling axis, contributing to the development of osteoporosis. b) Materials science and cross-applications: Research has expanded beyond traditional compounds to the development of innovative biomaterials. For example, Ye et al.^64^ (2023, cited 20 times) created a temperature-sensitive hydrogel infused with MnO_2_ nanoparticles that actively scavenges ROS in the osteoporosis microenvironment, thereby safeguarding transplanted stem cells and enhancing bone healing. Additionally, Shi et al.^73^ (2025, published online ahead of print, with official publication in 2026, cited 0 times) developed a platinum nanoframe designed for targeted delivery to bone tissue, which treats osteoporosis by simultaneously inhibiting the TMEM16A ion channel and scavenging ROS. c) Focus on new etiologies and targets: Research perspectives have broadened to include osteoporosis arising from various etiologies. For instance, Han et al.^74^ (2025) investigated diabetic osteoporosis and found that in a high-glucose environment, ROS contribute to osteocyte dysfunction and apoptosis by inhibiting autophagy and activating the mTOR signaling pathway. d) Exploration of novel compounds: There is a sustained effort to identify new active ingredients derived from natural products. For example, Qiu et al.^72^ (2025, cited once) reported that rosmarinic acid A mitigates ROS levels by activating the Nrf2-HO-1 pathway, thereby reducing osteoporosis-related bone loss. While some literature from 2025 has recently been published and has low citation counts, these research directions are innovative and have the potential for increased citation frequencies as they gain recognition.

In summary, research in this field has progressed from initially defining the fundamental role of ROS and established pathways to using multidisciplinary approaches that delve into complex mechanisms, create advanced materials, and broaden disease models. This evolution offers a wealth of innovative concepts and potential strategies for the prevention and treatment of osteoporosis.

#### Highly cited literature on other gas molecules and related factors

Among various signaling molecules, CO stands out as particularly significant. As a contributor to air pollution, chronic exposure to CO is regarded as a potential environmental risk factor for osteoporosis.^75^,^76^,^77^ A 2022 study highlighted the association between air pollution, specifically CO and nitrogen dioxide, and an elevated risk of osteoporosis,^75^ likely due to the synergistic interactions with other pollutants that impair bone health by inducing systemic inflammation and oxidative stress. Nevertheless, the independent and specific pathogenic molecular mechanisms of CO warrant further fundamental research for clarification. In a recent study by Yang et al.^78^ (2024, cited three times), a targeted upconversion nanoplatform for bone tissue was developed, capable of simultaneously storing and releasing two gas molecules, CO and H_2_S. By employing precise control with 808 nm near-infrared light, this platform modulates gas release within the osteoporosis microenvironment, achieving a multifaceted therapeutic effect: it promotes osteogenesis by activating osteoblasts, inhibits osteoclastogenesis by suppressing osteoclast activity, and enhances the immune microenvironment. High-throughput sequencing demonstrated that this platform effectively regulates inflammation, apoptosis, osteoclast differentiation, and various immune signaling pathways. This comprehensive treatment strategy, which aims to improve the bone microenvironment through the dual approach of promoting osteogenesis and inhibiting osteoclast activity, is a promising new avenue for addressing metabolic bone diseases.

## Discussion

### Summary and analysis of research findings

In this study, we conducted a bibliometric analysis of 387 publications regarding gas molecules and related factors associated with osteoporosis, sourced from the Web of Science Core Collection database, covering the period from 2001 to 2025. This comprehensive analysis outlines the developmental trajectory and current status of the field over the past 25 years. Our findings indicate a three-phase evolution in research: beginning with the association of phenomena, progressing to the elucidation of mechanisms, and culminating in the translation of materials into practical applications. The results suggest that research in this area is nearing a mature phase by around 2024, indicating a shift from a rapid growth stage to a more stable and mature phase. China leads in publication volume, contributing 170 papers, while the United States exhibits considerable academic influence with a higher average citation rate of approximately 66.^97^ citations per publication. The disparity in average citations between these two nations can be partially explained by their publication timelines; U.S. research was published earlier, enabling a longer window for citation accumulation. Notably, Chinese research tends to emphasize the intersection of natural product extracts and gas signaling, while U.S.-based studies often focus on the innovation of original nanoplatforms. This distinction accounts for the variation in citation averages. Together, both countries represent the primary driving forces in this research domain. Institutional analysis further supports this assertion, highlighting that Harvard University and its affiliated institutions, along with the Massachusetts Institute of Technology and leading medical universities in China, are at the forefront of exploration in this field.

The analysis of journal distribution reveals a predominant focus on “molecule-oriented and basic research,” with leading publications such as *Bone* and the *International Journal of Molecular Sciences* at the forefront, among a total of 244 source journals. This area of study continues to emphasize basic research, while clinical translational research remains relatively underdeveloped. Co-occurrence analysis of keywords reinforces the significance of “oxidative stress,” indicating “reactive oxygen species” (33 occurrences) and “oxidative stress” (31 occurrences) as not only high-frequency terms but also critical mechanistic nodes linking gas molecules and related factors to bone cell functions.

From the perspective of research content evolution, an analysis of keywords and highly cited literature reveals a distinct trajectory: early investigations primarily centered on defining the essential roles of oxidative stress and ROS in the pathology of osteoporosis, along with a preliminary examination of the bidirectional regulatory effects of classical gas molecules such as NO. In recent years, research has shifted towards a more in-depth exploration of underlying mechanisms, with a heightened focus on the role of H_2_S and endogenous antioxidant pathways, particularly the Nrf2 pathway. Moreover, the current research frontier has clearly transitioned towards clinical translational applications,^48^,^52^,^72^ with an emphasis on using nanotechnology and biomaterials engineering. Studies are increasingly focused on developing bone-targeted, controlled-release delivery systems for gas molecules (such as NO, H_2_S, and H_2_) to enable precise treatment of osteoporosis and its associated complications, including fractures.

### Key gas molecules and related factors in osteoporosis research

The paper primarily elucidates the fundamental roles of three types of gas molecules and related factors and their associated redox systems in the context of osteoporosis research, with ROS identified as the key pathological mediator. Recently, the Nrf2-related pathway has gained prominence as a significant area of mechanistic investigation.

NO, being the first gas-related factor to garner attention, has been the subject of ongoing research. Initial studies highlighted the critical mediating role of NO in inflammatory bone loss and its protective effects in relation to estrogen. However, contemporary research has progressed beyond basic mechanisms to encompass technological innovations, such as the development of near-infrared light-controlled release nanoplatforms and biomimetic scaffolds. These advancements aim to address the therapeutic challenges associated with the short half-life of NO and its lack of targeted delivery.

H_2_S has emerged as a prominent research focus over the past decade, transitioning from investigations into cellular protective mechanisms to the exploration of therapeutic applications. Recent studies have concentrated on developing bone-targeted nanodelivery systems, such as zeolitic imidazolate frameworks^52^ and hydrogel systems,^7^,^48^ with the goal of achieving precise and sustained release of H_2_S within bone tissue. This strategy aims to facilitate the healing of osteoporotic fractures by modulating macrophage immune metabolism and enhancing the coupling of osteogenesis and osteoclastogenesis, highlighting its considerable clinical translational potential.

H_2_, as an emerging medical gas, has become a frontier of exploration due to its selective antioxidant properties. Research has not only validated its bone-protective effects in animal models but has also advanced to developing smart biomaterials capable of releasing H_2_ (e.g., palladium-based nanocomposites)^53^ for local bone repair and regulating the immune microenvironment in osteoporosis. Additionally, the focus on the hypoxic microenvironment of bone tissue, along with studies on interventions such as hyperbaric oxygen, has expanded the dimensions of gas regulation in bone metabolism. provide nanother version

Emerging gas molecules and related factors, such as CO and H_2_S, have shown the potential to collaboratively enhance the bone microenvironment, stimulate osteoblast activity, and suppress osteoclast activity. Additionally, while ROS are not typical gas molecules, they are intricately associated with oxidative stress, functioning as a central pathological hub that integrates the effects of various gas molecules. As the keyword with the strongest correlation, ROS have been the subject of extensive research for over 20 years, beginning in 2002. Their mechanisms influencing osteoclast differentiation and inhibiting osteoblast function have been clarified through pathways such as RANKL, NF-κB, MAPK, and Nrf2/HO-1. The Nrf2/HO-1 pathway is emerging as a promising target for osteoporosis treatment, with the capacity to enhance the antioxidant defenses of bone tissue. Recent studies conducted between 2023 and 2025 have further illuminated the intricate regulation of the ROS/SIRT1/PGC-1α/P53 axis in cadmium-induced osteoporosis,^63^ as well as the pivotal role of mitochondrial ROS in age-related bone loss. This suggests a shift in the field from merely describing phenomena to engaging in a deeper exploration of underlying mechanisms.

In conclusion, the gas molecules discussed do not operate independently; rather, they create a complex regulatory network, with the primary focus being the modulation of intracellular reactive oxygen levels and oxidative stress. This regulation significantly influences the functions, differentiation, and apoptosis of osteoblasts, osteoclasts, and osteocytes, acting as a central hub that connects various pathological mechanisms associated with osteoporosis. The developmental trends and translational potential of the three key gas molecules (NO, H_2_S, and H_2_) as well as other crucial factors (such as ROS and the Nrf2/HO-1 pathway) are summarized in **Table 7**.

**Table 7:** Summary of the developmental stages and characteristics of gas molecules and related factors in osteoporosis research

Gas molecules and related factors	Research stages	Core focus and representative progress	Trends
NO	Early foundations and recent transformations (2002 to present)	Classic mechanism: Establishing the core role of NO in oxidative stress and inflammatory bone loss, and validating the bone protective effects of NO donors (such as nitroglycerin). Latest frontiers: Developing bone-targeted nano-delivery systems (such as near-infrared light-triggered release) and multifunctional biomimetic scaffolds to achieve controlled and precise release of NO in the local bone environment.	The research focus has shifted from exploring pathophysiological mechanisms to innovations in delivery technologies. Efforts are dedicated to overcoming clinical translation bottlenecks related to the short half-life of NO and the lack of targeting.
H_2_S	Rapid rise and current hot topics (2014 to present)	Mechanism establishment: Clarifying the antioxidant protective effects of H_2_S on osteoblasts and its mechanisms in regulating bone metabolism. Engineering breakthroughs: Designing bone-targeted H_2_S nano-delivery systems (such as zeolitic imidazolate frameworks) that can co-release therapeutic ions, promote healing by reprogramming immune cell metabolism, and regulate the osteoblast-osteoclast coupling.	The research has quickly transitioned from molecular mechanism studies to the development of advanced formulations. This is currently the most dynamic and clinically relevant research direction in the field.
H_2_	Emerging frontiers (recently emerged, median year 2023)	Preliminary validation: It has been discovered that H_2_ or H_2_-rich water can selectively inhibit osteoclasts through antioxidant mechanisms, preventing bone loss in animal models. Material integration: Developing injectable H_2_-releasing biomaterials (such as magnesium-loaded hydrogels) that continuously generate H_2_ at the affected site to scavenge ROS and modulate the immune microenvironment.	The research is in the early validation stage but has rapidly integrated with biomaterials and tissue engineering strategies, representing a new expansion in gas therapy.
ROS	Classic pathological mechanisms and ongoing hot topics	Core role: As a key driving factor in the pathogenesis of osteoporosis, excessive production of ROS leads to oxidative stress, impairing osteoblast function, accelerating osteocyte apoptosis, and promoting osteoclast activity, ultimately resulting in bone loss. Pathway research: It has been elucidated that ROS affect the balance of bone formation and resorption by interfering with important signaling pathways such as FoxO and Wnt. In osteoporotic fractures, oxidative stress also accelerates the apoptosis of osteoblasts and osteocytes, hindering the healing process.	Research has shifted from elucidating pathological effects to exploring intervention strategies. The current focus is on how to mitigate bone loss by regulating ROS levels or activating endogenous antioxidant pathways (such as Nrf2/HO-1).
Nrf2/HO-1 pathway	Mechanism clarification and targeted exploration (recent hot topics)	The Nrf2/HO-1 pathway is the intracellular core antioxidant defense axis. Activation of this pathway can inhibit ROS, alleviate oxidative stress and inflammation, thereby promoting osteogenesis and inhibiting osteoclastogenesis. Numerous active components of traditional Chinese medicine exert bone protective effects through this pathway.	Research has shifted from basic mechanistic studies to the development of specific pathway activators (natural products/synthetic compounds) as therapeutic strategies.

NO: Nitric oxide; Nrf2/HO-1: nuclear factor erythroid 2-related factor 2/heme oxygenase 1; ROS: reactive oxygen species.

### Limitations

The bibliometric analysis presents several limitations: (1) The analysis relies exclusively on citation information from the Web of Science Core Collection, omitting literature from other significant databases such as Scopus, PubMed, and CNKI. (2) Publications from 2024–2025 may not achieve adequate citation frequency to accurately represent their academic value due to the limited citation timeframe. (3) Most of the current studies are preclinical, involving animal models and cell experiments, which results in a notable deficiency of clinical trials, thereby restricting the applicability of the findings to clinical practice. (4) There may be an underestimation of data from 2024-2025, particularly regarding citation frequency, due to delays in literature indexing and citation tracking.

### Study significance and conclusions

The study offers a thorough bibliometric and visual analysis of research on gas molecules and related factors in osteoporosis from 2001 to 2025, emphasizing both theoretical and practical significance. It effectively maps the field’s complete evolutionary trajectory, transitioning from “phenomenon association” to “mechanistic insights” and finally to “material translation.” The study outlines the various research stages and evolving hotspots for different gas molecules, providing a comprehensive overview that enables future researchers to quickly understand the landscape and identify research gaps. Current research frontiers and breakthroughs focus on the development of advanced delivery systems, highlighting opportunities for cross-disciplinary innovation in biomaterials, pharmacy, and bone science. This suggests that a major trend in future strategies for addressing osteoporosis will involve designing smart, targeted gas-releasing materials, which has significant practical implications. Consequently, gas molecules and related factors, particularly NO, H_2_S, and H_2_, along with their associated redox regulation, have emerged as crucial areas in both basic research and therapeutic development for osteoporosis, in line with recent literature findings. Research in this field has entered a new phase characterized by precise targeted delivery and material innovation in translational medicine.^79^ While basic research has reached a relatively advanced stage, its deep integration with fields such as nanotechnology and biomaterials engineering offers significant potential for innovation and wide-ranging prospects for clinical translation.

### Future suggestions

While promising research results have emerged in this field, clinical validation remains in its early stages. The translation of findings from preclinical research to clinical applications currently faces several challenges, including dosage control of gas molecules, long-term biocompatibility assessments, and the selection of appropriate clinical endpoints for osteoporosis. Future research should not only focus on utilizing new materials for delivering gas molecules but also clarify their specific target actions and spatiotemporal dynamics within the complex bone microenvironment, which comprises blood vessels, nerves, and immune cells. This understanding will provide a theoretical foundation for optimizing designs. Moreover, future gas therapy strategies should extend beyond the delivery of single molecules to develop integrated smart platforms that combine multiple gas molecules (such as the synergistic effects of NO and H_2_S) or integrate them with other therapeutic agents (such as growth factors, metal ions, or drugs) to achieve synergistic effects. Currently, preclinical studies predominantly focus on postmenopausal osteoporosis. Future efforts should enhance the validation of gas therapy strategies for other types of osteoporosis, such as glucocorticoid-induced, senile, and disuse osteoporosis, while actively progressing promising gas therapy strategies toward early clinical trials. The dose-response relationship of gas molecules is complex, and high local concentrations may lead to toxicity. Therefore, it is crucial to systematically evaluate the long-term biocompatibility, metabolic pathways, and potential adverse reactions of new materials. Continuous advancements in this field depend on the deep integration of bone biology, gas biology, nanomedicine, biomaterials engineering, and clinical medicine. Establishing closer interdisciplinary teams is encouraged to collaboratively address the barriers from basic research to clinical application. In addition to the existing focus on nanodelivery systems, future research could enhance investigations into the synergistic effects of various gas molecules, precise spatiotemporal control of gas release, and the mechanisms and treatment explorations for specific types of osteoporosis, including diabetic and disuse osteoporosis.

## Data Availability

*Not applicable.*
